# Molecularly Imprinted Polymers and Magnetic Molecularly Imprinted Polymers for Selective Determination of Estrogens in Water by ESI-MS/FAPA-MS

**DOI:** 10.3390/biom10050672

**Published:** 2020-04-27

**Authors:** Maria Guć, Grzegorz Schroeder

**Affiliations:** Faculty of Chemistry, Adam Mickiewicz University in Poznan, Uniwersytetu Poznańskiego 8, 61-614 Poznań, Poland; schroede@amu.edu.pl

**Keywords:** mag-MIPs, MIPs, E1, E2, hormones, separation techniques, mass spectrometry, ESI-MS, FAPA-MS, environment contamination

## Abstract

Qualitative and quantitative analysis of estrogens content in natural water is a difficult task. An important problem in the analysis of hormones in water is the quantitative determination of their individual species. Low detection limits and instability of estrogen derivatives are the main challenges. Magnetic molecularly imprinted polymers (mag-MIPs) in combination with Flowing Atmospheric-Pressure Afterglow Mass Spectrometry (FAPA-MS) were successfully used for analysis of estrogen hormones in water samples. The aim of the study was to obtain mag-MIPs selective to estrone (E1) and β-estradiol (E2) for solid phase extraction and pre-concentration of estrogens. Due to their superior analyte binding properties at low concentrations (0.03 g in 1 g of polymer structure) and possibility of magnetic separation, mag-MIPs were proven to be very convenient and efficient adsorbent materials. In addition, MS analyses were performed using two ionization sources: ESI- and FAPA-MS. For both estrogens, LOD was significantly lower for FAPA-MS analysis (0.135 μg L^−1^ for E1 and E2) than for ESI-MS analysis (27 μg L^−1^ for E1 and 13.6 μg L^−1^ for E2). The total estrogen concentration in the environmental water sample was determined as: cE1 = 0.271 μg L^−1^ and cE2 = 0.275 μg L^−1^.

## 1. Introduction

In recent years, emerging contaminants such as steroid sex hormones, which are referred to as endocrine disruptors or endocrine disrupting compounds, have become of particular concern due their increased use and physiological activity. Both natural and synthetic steroid hormones can be found in the environment, and they are believed to enter water systems with farming and household waste. Hormones are substances synthesized by specialized glands [1]. They act as signaling compounds, regulating various cellular processes and modifying structural features of target tissues. Due to their high biological activity, in vivo estrogens are produced at low concentrations. In general, hormones belong to different classes of chemical compounds. There are several dozen steroid hormones that fulfill a wide range of functions in plants, animals, and humans [2]. This research was focused on selected hormones from the estrogen group. The presence of endocrine disrupting chemicals in water has gained global attention, as they may cause adverse effects in aquatic ecosystems and human body even at low concentrations. Endocrine disrupting compounds contain a wide range of chemicals, besides steroid hormones, including a group of biologically active compounds that are synthesized from cholesterol and have a similar structural characteristics such as a cyclopentano-perhydrophenanthrene ring. The natural estrogens (Table S1), E1, E2, and estriol (E3), and the synthetic one, 17α-ethinylestradiol (EE2), are most commonly found in wastewater [1,3]. The results of a recent research on aquatic ecosystems have caused major concerns regarding increased occurrence of such phenomena as feminization of individuals and hermaphroditism. Unfortunately, determination of natural and synthetic estrogens in water is a difficult analytical task [4]. Estrogens E1, E2, and E3 lie on inter-connecting metabolic pathways. It has been demonstrated that microorganism living in aerobic conditions are capable of converting one estrogen into another. Additionally, many studies indicate that E2 is the main degradation product of E1.

Furthermore, the half-life of an estrogen depends on its rate of degradation. The half-life can be determined by the first-order decay or regression curve kinetics, whereas the rate of degradation can be estimated directly from decay kinetics. Clearly, the longer is the half-life of a pollutant, the more persistent it is in the environment. Estrogens that are excreted by humans and animals have short half-lives. Due to a mainly hydrophobic character, their concentrations decrease significantly in the aqueous phase. In work by Silva et al. [3], estrogen half-lives were calculated in range from 48 min to 6 days in water and different environmental samples. Xiao et al. [5] and Briciu et al. [6] reported that degradation of E2 and E1 occurred in rivers, lakes, and sludge with a half-life of 0.2–9 days at 20 °C. The main parameters determining estrogens stability in aqueous solutions are: pH of the environment, temperature, oxygen content, and the presence of aerobic microorganism [7,8]. 

Molecular imprinting, which was established in 1972 by Wülff and Sarhan, is an attractive approach to generate artificial receptors [9].

**Figure 1:**
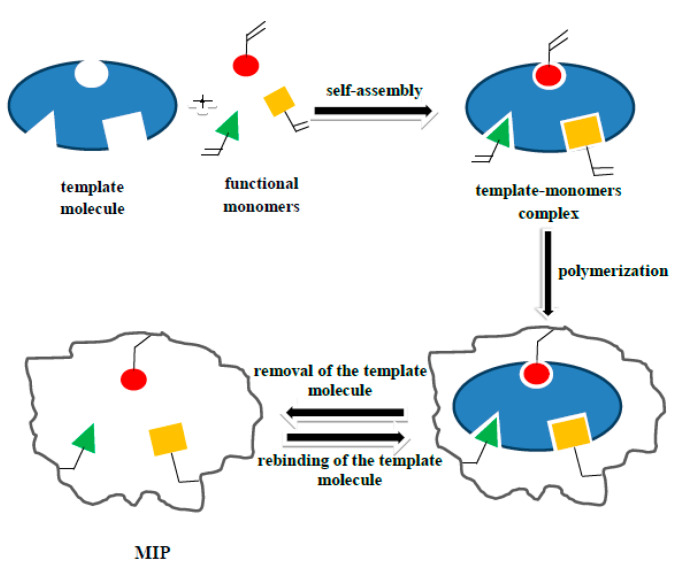
General scheme of obtaining MIP.

The technology of MIPs (Figure 1) and mag-MIPs production is based on the formation of complexes between the analyte/template/guest molecule and the functional monomer/host molecule. The technique involves polymerization of functional monomers and a cross-linker around a template [10]. The analyte-monomer complex is then subjected to polymerization initiated by temperature or irradiation (thermal or photoinitiation), during which a three-dimensional polymer structure is formed. At the subsequent stage, the template is removed from the polymeric structure, leaving empty cavities, recapitulating its spatial signature (size, shape, and functional groups positioning) and capable of binding molecules identical to the template. The use of organic porogens makes the polymer hydrophobic. The polymers obtained are chemically stable and water insoluble, which permits multiple uses in chemical analysis. Additionally, their synthesis process is relatively simple, reproducible, and cheap [11,12]. MIPs have previously been used for the analysis of pharmaceutical compounds, estrogens, and other steroids in surface and wastewater. The new core-shell synthesis of mag-MIPs, in which molecularly imprinted polymer surface (shell) covers magnetic iron oxide nanoparticles (core) [13], receives widespread attention [14,15]. After the synthesis, the material retains its magnetic properties, and thus can be easily manipulated using a neodymium magnet, while providing selectively adsorptive capabilities. Additionally, the decreased size of the mag-MIP particles increases the surface area, enhancing the activity per unit mass of the polymer. Moreover, the recognition sites are located on the surface of the material, facilitating the access of the analyte to the selective cavities, and its easy removal. In comparison to the MIP obtained by traditional synthesis methods, mag-MIPs present a wide set of advantageous properties [16,17]. Due to the large number of recognition sites located on the surface of MIPs in each mag-MIPs particle, the quantification of analytes using these materials is expected to be more sensitive and selective [18,19].

Ambient ionization mass spectrometry comprises a group of various techniques which allow MS analysis at atmospheric pressure. They provide rapid, direct, and high-throughput analyses with no or only minimal sample pre-treatment. Moreover, substances can be analyzed directly from surfaces or other matrices. FAPA-MS techniques involve the generation of a direct current or radiofrequency electrical discharge between a pair of electrodes in contact with a flowing inert gas, creating a stream of ionized molecules, radicals, excited state neutral atoms, and electrons. The plasma species are directed towards the sample, resulting in desorption and ionization of the analyte. Ambient plasma MS techniques have many advantages due to their simple instrumentation, rugged construction of the measuring system, no solvents requirement, and generation of singly charged analyte species that are more easily identifiable than multiple charged ions and various adducts, produced by spray-based techniques. This work presents the analytical procedures for determination of selected estrogens (E1 and E2) in water solutions with the use of MIPs or mag-MIPs, for pre-concentration and stabilization, and FAPA-MS with thermal desorption of analytes. The proposed method is compared with the direct determination of estrogens in water solutions based on ESI-MS [20,21,22].

## 2. Materials and Methods 

### 2.1. Chemicals 

All reagents used were commercial products. The compounds FeCl_2_·4H_2_O, FeCl_3_·6H_2_O, tetraethoxysilane (TEOS), hydrochloric acid (HCl), citric acid, sodium hydroxide (NaOH), ammonia solution (NH_4_OH), methacrylic acid (MA), ethylene glycol dimethacrylate (EGDMA), 2,2′-azobisisobutyronitrile solution 0.2 M in toluene (AIBN), Aluminum Oxide Activated, basic, Brockman I, 3-(trimethoxysilyl)propyl methacrylate (MPS), E1, E2, and all solvents (toluene, ethanol, and acetic acid of the purity grade p.a.) were obtained from Sigma-Aldrich (St. Louis, MO, USA).

### 2.2. Synthesis 

#### 2.2.1. E1- or E2-MIP

MIPs were synthesized with a method similar to that described in literature [23]. First, 1 mmol of the template molecule E1 or E2 and 4 mmol of the functional monomer-MA were dissolved in 30 mL ethanol. Subsequently, 20 mmol of the cross-linking agent-EGDMA were added (additionally purified with basic Activated Aluminum Oxide, Brockman I). The ratio of the template:monomer:cross-linking agent was 1:4:20. The pre-polymerization solution was sonicated and purged with nitrogen for 30 min. Afterwards, 1.25 mL of initiator-AIBN was added. The tube was sonicated and purged with nitrogen for an additional 40 min, sealed, and placed in an oven for 24 h at 60 °C. After polymerization, E1- or E2-MIP was dried under reduced pressure and grounded using mortar and pestle. The particles of E1 or E2 were extracted with a mixture of ethanol and acetic acid (9:1, *v:v*) via Soxhlet extraction for 90 h. Lastly, the MIPs were dried at 60 °C under vacuum and grounded.

#### 2.2.2. E1- or E2-Mag-MIP

Mag-MIPs were synthesized with a method similar to that described in literature [24]. 

##### Magnetic Core

The magnetite nanoparticles (Fe_3_O_4_) were synthesized in a two-step procedure. Firstly, magnetite (Fe_3_O_4_) was synthesized by using FeCl_2_·4H_2_O and FeCl_3_·6H_2_O as precursors. Secondly, the surface-functionalized magnetite was produced via hydrolysis and condensation of TEOS organosilane agent. FeCl_2_·4H_2_O (2 g) and FeCl_3_·6H_2_O (5.2 g) were dissolved in 25 mL of deoxygenated water, and then 0.85 mL of concentrated HCl were added. The resulting solution was added dropwise into 250 mL of 1.5 M NaOH solution upon vigorous stirring and N_2_ protection at 80 °C. The synthesized magnetic nanoparticles (MNPs) were separated from the solution with a neodymium magnet and washed with 200 mL of deionized water three times. Obtained Fe_3_O_4_ nanoparticles were further stabilized for the preparation of an aqueous suspension. A total of 10 mL of an aqueous solution of citric acid (0.5 g mL^−1^) was added to a vigorously stirred suspension of washed nanoparticles. The pH value was set to 5.2 with a concentrated ammonia solution and heated to 80 °C. After 90 min, the pH value of the solution was elevated to 10. Lastly, the suspension was centrifuged for 5 min at 4000 rpm to remove any agglomerated nanoparticles. Next, silica coat was fabricated on the surface of Fe_3_O_4_ MNPs through a sol-gel method. One hundred milliliters of ethanol containing 4 mL TEOS were added to the above prepared stable aqueous suspension of Fe_3_O_4_ MNPs, followed by stirring at ambient temperature for 6 h. After rinsing with ethanol and water several times, the obtained Fe_3_O_4_@SiO_2_ was dried at 60 °C. In the next step, Fe_3_O_4_@SiO_2_ was modified with MPS. For this purpose, 250 mg of this material was dispersed in 50 mL of anhydrous toluene containing 5 mL of MPS and the mixture was allowed to react at 60 °C for 24 h under nitrogen atmosphere. The mixture was filtered through a membrane, washed with toluene, and dried in vacuum. Fe_3_O_4_@SiO_2_-MPS was obtained. 

##### Polymer Shell

Mag-MIPs were prepared via polymerization of 1 mmol template-E1 or -E2 and 4 mmol functional monomer-MA in 30 mL of a porogenic solvent-ethanol. The mixture was placed in ultrasound bath at 25 °C for 1 h, and then 200 mg of Fe_3_O_4_@SiO_2_-MPS were added into the system. The mixture was placed in ultrasound bath for 1 h. Subsequently, 20 mmol of the cross-linking agent-EGDMA and 1 mL of the initiator-AIBN were added into the system and the mixture was sonicated at 80 °C for 24 h. After polymerization, E1- or E2-mag-MIP was dried under reduced pressure and grounded using mortar and pestle. Afterwards, the template was removed after polymerization via Soxhlet extraction using a ratio of ethanol:acetic acid (9:1, *v:v*) as eluent, which was replaced every 90 h. The obtained products were dried and grounded. 

The elimination of the E1/E2-template was assessed with two techniques. The template concentration in washing solution was tested with ESI-MS^2^. The concentration of the template in the dried MIPs and mag-MIPs was tested with FAPA-MS^2^. After the extraction, the obtained materials contained about 1% and 4% of estrogens in the MIP and mag-MIP, respectively. Further extraction or solvent exchange did not facilitate further template removal.

#### 2.2.3. NIP and Mag-NIP

As a control experiment, non-imprinted polymer (NIP) and magnetic non-imprinted polymer (mag-NIP), without a template addition during the polymerization, were also prepared and treated in an identical manner as MIPs and mag-MIPs.

### 2.3. Instruments 

The infrared spectra analyses were performed on IFS 66 v/s Fourier Transform Infrared (FT-IR) spectrophotometer (Bruker), equipped with MCT detector (125 scans, resolution 2 cm^−^^1^). The spectra were recorded in the 400–4000 cm^−^^1^ range for KBr pellets. To confirm successful functionalization, the materials were subjected to thermogravimetric analysis (TGA). The measurements were performed on Setsys 1200 thermogravimetric analyzer (Setaram) at heating rate of 10 °C min^−^^1^ starting from room temperature to 1000 °C under helium atmosphere. Scanning electron microscopy (SEM) images were acquired with QUANTA 250 FEG, FEI. ESI-MS and ESI-MS^n^ spectra were recorded using amaZon SL ion trap (Bruker, Bremen, Germany) equipped with electrospray ion source operating in infusion mode. The sample solution was introduced into the ionization source at a flow rate of 5 μL min^−1^ using a syringe pump. The apparatus was operated using “enhanced resolution mode” (mass range: 50–2200 *m/z;* scanning rate: 8,100 *m/z* per second). The capillary voltage was set at −4.5 kV and the endplate offset at −500 V. The source temperature was 80 °C and the desolvation temperature was 250 °C. Helium was used as the cone gas and desolvating gas (nitrogen) at flow rates of 50 and 800 Lh^−1^, respectively. The mass spectrometer was operated in the ESI positive and negative ionization mode. In MS^n^ experiments, the width of the selection window was set at 2 Da and the amplification of the excitation was set according to the experiment (from 0.2 to 1.5 V). Mass spectrometers were equipped optionally with FAPA ambient plasma source (ERTEC, Wroclaw, Poland). The experimental details of FAPA method are presented in previous publications [25,26,27]. 

### 2.4. Analysis 

#### 2.4.1. Calibration Curve

Aqueous solutions of E1 and E2 were prepared in the concentration range from 0.27 μg L^−1^ to 2.70 mg L^−1^. ESI-MS spectra were recorded. Analytical signals were selected, and then the relationship between signal intensity and estrogen concentration was plotted.

#### 2.4.2. Estrogens Degradation in Water as a Function of Time

Aqueous solutions of E1 and E2 were prepared in two concentrations (5.5 and 1.35 mg L^−1^). Spectra were recorded for all 4 samples over time: 5, 10, 20, 30, 60, and 120 min, 24 h, and 7 days. The relationships of E1 and E2 concentrations versus time were plotted.

#### 2.4.3. E1-MIP, E2-MIP, E1-Mag-MIP and E2-Mag-MIP Selectivity for E1 or E2 in Hormone Aqueous Solutions

In the first series, 10 mg E1-MIP or E1-mag-MIP was added to 10 mL of E1 aqueous solution and to 10 mL E2 aqueous solution. Four different mixtures were obtained. In the second series, 10 mg E2-MIP or E2-mag-MIP was added to 10 mL of E1 or E2 aqueous solution. The concentration of E1 or E2 in aqueous solutions was 2.7 mg L^−1^. All eight mixtures were shaken for 2 h, and then the sediments were centrifuged or isolated using a neodymium magnet. The solutions were analyzed via ESI-MS. The spectra of hormone solutions recorded immediately after preparation (*t* = 0 h) were used as the reference point. 

#### 2.4.4. The Effect of pH on Estrogens Release from E1-Mag-MIP and E2-Mag-MIP

For this purpose, 10 mg of polymers containing bound analyte were added to 10 mL of the buffer solutions with pH of: 5.0, 7.0, and 9.5. Hormone concentrations in buffer solutions were measured after 10, 20, 30, 40, 50, and 60 min, using the ESI-MS method. 

#### 2.4.5. Determination of Estrogens in Water Using E1-Mag-MIP and E2-Mag-MIP Combining with FAPA-MS Method

Portions of 10 mg of appropriate mag-MIPs (E1-mag-MIP or E2-mag-MIP) were added to 10 mL of aqueous solutions containing estrogens. The solutions with mag-MIPs were stirred for 5 min. Then, E1-mag-MIP and E2-mag-MIP were isolated using a neodymium magnet and subsequently dried in the open air. Portions of 10 mg of polymers with adsorbed analyte were placed on the apparatus heating table, heated, and measured with the FAPA-MS method. 

## 3. Results and Discussion

Pharmaceuticals most frequently identified in the aquatic environment include beta-blocker drugs, non-steroidal anti-inflammatory drugs, antibiotics, and female sex hormones [28]. Sex hormones include natural estrogens E1, E2 and E3 used in hormone replacement therapy and as a component of contraceptives. In the rivers of Europe, the estimated concentration of these hormones is 0.3–3.5 ng L^−^^1^ [29,30] Due to their high biological activity, it is necessary to develop analytical methods that would allow determination of their concentration not only as a sum of estrogens, but also of particular species, E1 or E2. The solubility of estrogens in water ranges from 0.8 to 30 mg L^−^^1^ (Table S1).

### 3.1. Synthesis 

**Figure 2:**
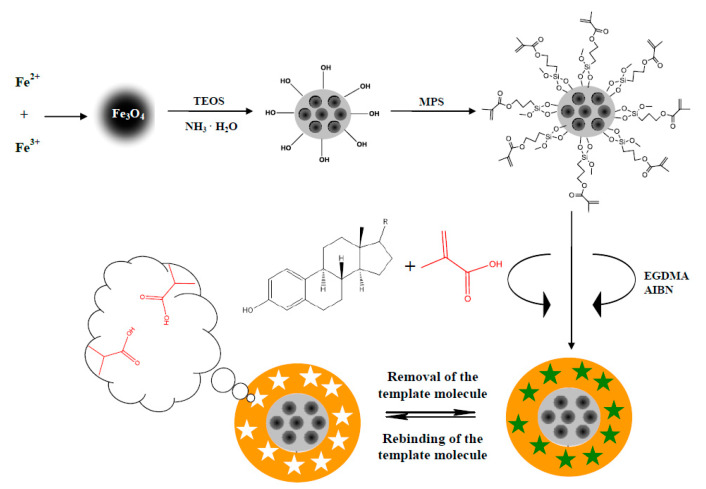
General steps for the preparation of estrogens (R = O or OH) magnetic molecularly imprinted polymers.

The polymers were obtained via the synthesis of E1- and E2-MIP (Figure S1) and their magnetic analogs E1- and E2-mag-MIP (Figure 2) as well as NIPs/mag-NIP without cavities formed by the addition of templates. 

As obtained MIPs/mag-MIPs are selective towards E1 and E2. Empty cavities of MIPs/mag-MIPs containing carboxyl groups and a suitable spatial structure differentiate the binding of estrogens. E2 contains -OH group, forming strong hydrogen bond with -COOH group of the polymer. 

### 3.2. Analysis 

#### 3.2.1. FT-IR Spectra 

At each stage of the synthesis, structure of the products was examined with FT-IR method. The obtained magnetic and non-magnetic adsorbent materials differ in their internal structure. Mag-MIPs/mag-NIPs are core–shell materials with a Fe_3_O_4_@SiO_2_ core, while MIPs/NIPs are coreless polymers also used as the outer shell. In the FT-IR spectrum of pure Fe_3_O_4_, the Fe-O bond absorption band appeared at 580 cm^−1^. The characteristic absorption of bare Fe_3_O_4_ for Fe_3_O_4_@SiO_2_ was observed at 576 cm^−1^ (Fe-O vibrations). The spectrum of Fe_3_O_4_@SiO_2_ showed strong peaks at 1085 and 796 cm^−1^. These peaks could be assigned to the asymmetric and symmetric linear stretching vibrations of the Si-O-Si bonds. The bending vibration absorption peaks of Si-O-Si and Si-OH were observed at 462 and 962 cm^−1^, respectively. These are indicative of the silica layer on Fe_3_O_4_. However, these bands were not detected for the core–shell materials. This is understandable, as, in the FT-IR analysis, signals from the surface of a material are more prevalent. The MIPs/NIPs and the core–shell materials have the same polymeric surface; therefore, their FT-IR spectra are analogous. 

**Figure 3:**
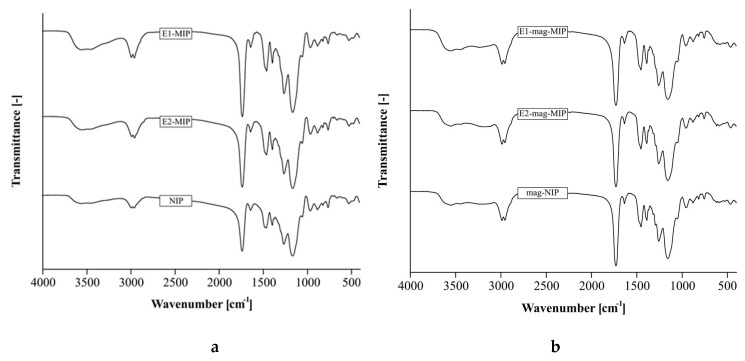
The FT-IR spectra of: (**a**) E1-MIP, E2-MIP and NIP; (**b**) E1-mag-MIP, E2-mag-MIP, and mag-NIP.

FT-IR analyses of E1-MIP, E2-MIP, NIP (Figure 3a), E1-mag-MIP, E2-mag-MIP, and mag-NIP were performed (Figure 3b). The majority of the signals can be attributed to the polymeric surface of the materials. After polymerization, C=C double bond stretching (1635 cm^−1^) and C=O double bond bending (555 cm^−1^) signals were observed and ascribed to the double bond in the MAA monomer. Moreover, the O-H stretching at 2935 cm^−1^ and the O-H bending vibration at 1383 cm^−1^ confirmed the presence of carboxylic groups. The presence of peaks at 1717 (C=O stretching) and 1140 cm^−1^ (C-O stretching) show the existence of EGDMA cross-linker. The C-O-C asymmetric and symmetric group signals at 1256 and 1041 cm^−1^ can be derived from the monomer chain. The stretching vibrations at 1294–1293 cm^−1^ for C-O bond and stretching vibrations at 1076 cm^−1^ for C-H bonds are characteristic of these groups. Asymmetrical stretching absorption at 655–650 cm^−1^ for C-O-C, bending vibration at 626–622 cm^−1^ for C-O-H, and bending absorption at 566–564 cm^−1^ for C-C=O are observed in the spectra.

The NIP/mag-NIP and MIPs/mag-MIPs spectra showed only slight differences in the intensity and positioning of the signals. The hydrogen bond and the van der Waals interactions between polymers and templates resulted in observable, small changes in spectra at 2700–3700 cm^−1^ range. 

A strong and broad absorbance peak assigned to the stretching vibration of hydroxyl groups was found at 3300 cm^−1^. Hydroxyl groups in the hormone and monomer molecule formed intra- and/or inter-molecular hydrogen bonds O-H. In general, steroidal estrogens containing only a phenolic hydroxyl, e.g., E1, produced an O-H band at a higher wavelength than those containing only an alcoholic hydroxyl, e.g., E2 [31,32,33]. The least intense band from hydrogen bonds is observed in NIP and mag-NIP reference samples, stemming from the monomer interactions in the polymer structure. These results show that carbonyl and hydroxyl groups are the dominant functional groups in the imprinted polymer and play a crucial role in the interactions between templates and polymers. 

#### 3.2.2. Thermal Analysis 

**Figure 4:**
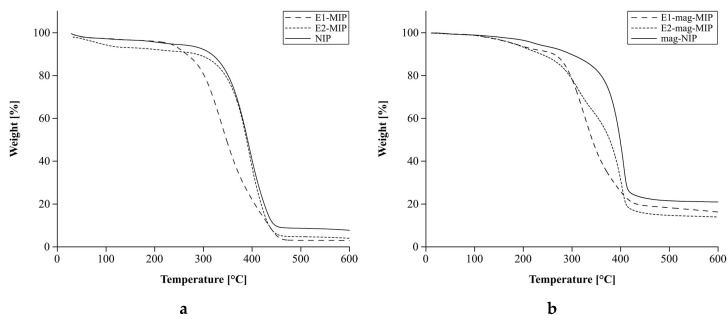
Thermogravimetric curves of: (**a**) E1-MIP, E2-MIP, and NIP; (**b**) E1-mag-MIP, E2-mag-MIP, and mag-NIP.

Thermogravimetric curves of the materials studied are shown in Figure 4a,b.

The polymers are stable to about 100 °C. For E2-MIP, the loss of mass was observed at lower temperature, which is related to its melting point, 178 °C for E2. The melting point of E1 is slightly higher and amounts to 260 °C. Therefore, around this temperature, a loss of mass for E1-MIP was observed. It confirms that during the process of polymer synthesis, the templates were stable. We successfully obtained polymers with various cavities binding E1 or E2 molecules. The greatest mass loss was observed in the range 180–300 °C, and it was more pronounced for E1-/E2-MIP and E1-/E2-mag-MIP than for NIP and mag-NIP. It is interpreted as the release of hormones from the materials’ structures. In the range of 178–300 °C the most intensive desorption of E2 was observed. For E1, the range was 260–300 °C. These ranges, 178–300 °C for E2 and 260–300 °C for E1, were applied for thermal desorption of the analyte from E1-/E2-MIP and E1-/E2-mag-MIP in FAPA-MS analysis. Twenty percent of residual material in the case of magnetic polymers may result from the presence of a magnetic core that has not decomposed within the temperature range.

#### 3.2.3. SEM Images 

All of the obtained materials were characterized with SEM. The SEM images (low (500×) and high (30,000×) magnification) are shown in Figure S2. It is visible that the surface of E1-MIP/E2-MIP after the template removal is more homogenous in comparison the polymer before the template removal. Images reveal that the obtained magnetic particles have a spherical morphology and a diameter less than 100 nm. Due to their small size, these nanoparticles agglomerate easily. Moreover, the presence of the core ensures specific particle size and shape. The polymer builds up around the spherical core to form spherical particles whose area is greater than that of MIPs. Moreover, the recognition sites are located on the surface of the material, facilitating the analyte binding. Due to their magnetic and surface properties, mag-MIPs are more efficient than MIPs obtained by traditional synthesis methods, as indicated in Section 3.2.5. These advantages of mag-MIPs render them highly attractive for a wide variety of applications in separation techniques. 

#### 3.2.4. Analysis of Estrogens in Aqueous Solution Using the ESI-MS Technique 

The studies were carried out for aqueous solutions of E1 and E2 in the range from 0.27 μg L^−1^ to 2.7 mg L^−1^. In the ESI-MS analysis (positive ions mode) of the freshly prepared E1 solution, a signal was observed at *m/z* 271 [M+H]^+^ and a low intensity fragmentation signal *m/z* 253 [M-18]^+^ (Figure S3). This mode of fragmentation of the E1 molecule was confirmed by the ESI-MS^2^ spectra (Figure S4). In the case of E2, we observed a signal at *m/z* 273 [M+H]^+^ and a fragmentation signal at *m/z* 255 [M-18]^+^ (Figure S5). This fragmentation pathway of the E2 molecule was confirmed by the ESI-MS^2^ spectra (Figure S6). The relationship between the signal intensity and the concentration of analyte was plotted (Figure S7). It was found that, for the samples prepared immediately prior to the measurement, the range of linearity in the E1 and E2 determination ranged from 0.135 to 2.7 mg L^−1^, while the LOD of E1 and E2 in the ESI-MS method was 27 and 13.6 μg L^−1^, respectively. 

**Figure 5:**
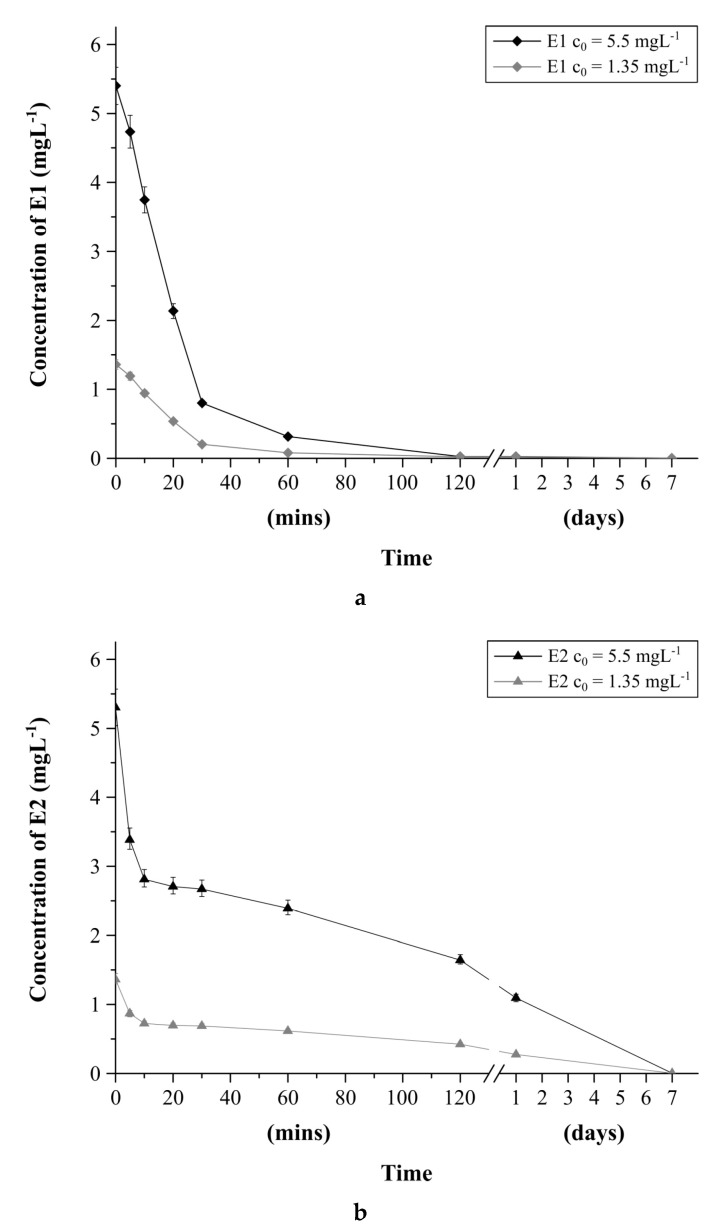
(**a**) Concentration of E1 in aqueous solution as a function of time. (**b**) Concentration of E2 in aqueous solution as a function of time.

Under the influence of environmental factors (pH, temperature, and oxygen), estrogens undergo chemical transformation. The rate of this process in water is strongly dependent on the concentration of hormones. In laboratory conditions, the stability of E1 and E2 in water at pH = 7 was tested (Figure 5b) as a function of time: measurements were performed after 5, 10, 20, 30, 60, 120 min, 24 h, and 7 days for two concentrations of each of the hormones: 5.4 and 1.35 mg L^−1^.

E1 signal gradually disappeared in water samples, which was related to its inter-conversion into E2. The concentration drop was the most pronounced up to 30 min after the dissolution of E1 in water at pH = 7. After 120 min, its concentration remained constant. The concentration of E1 in water after this time was similar, 24 μg L^−1^ for both initial concentrations of E1 studied. This value decreased slightly after 24 h. It can be concluded that, in solutions with high dilutions of estrogens, compounds degrade more slowly. However, after a week, no signals from E1 were detected. The concentration of E2 was most intensively reduced in up to 10 min after its dissolution in water. Then, the concentration of E2 decreased slowly in direct proportion to the initial concentration. The E2 concentration after 120 min was 1.64 mg L^−1^ for the higher initial concentration and 0.41 mg L^−1^ for the lower one. Within 24 h, the concentrations slightly decreased, but, after a week, the signals from E2 were also not detected. The rate of decomposition of estrogens in water is an additional factor hindering the determination of this group of compounds. The development of selective MIPs/mag-MIPs, and their use for pre-concentration in chemical analysis using various mass spectrometry techniques, allows the development of a new analytical procedure for the qualitative and quantitative determination of E1 and E2 in aqueous solutions. Furthermore, the use of MIPs/mag-MIPs is a simple and effective method for stabilizing and storing analytes. Binding of E1 and E2 in polymer structures prevents their conversion and degradation.

#### 3.2.5. E1-MIP, E2-MIP, E1-Mag-MIP and E2-Mag-MIP Selectivity for E1 or E2 in Hormone Aqueous Solutions 

**Figure 6:**
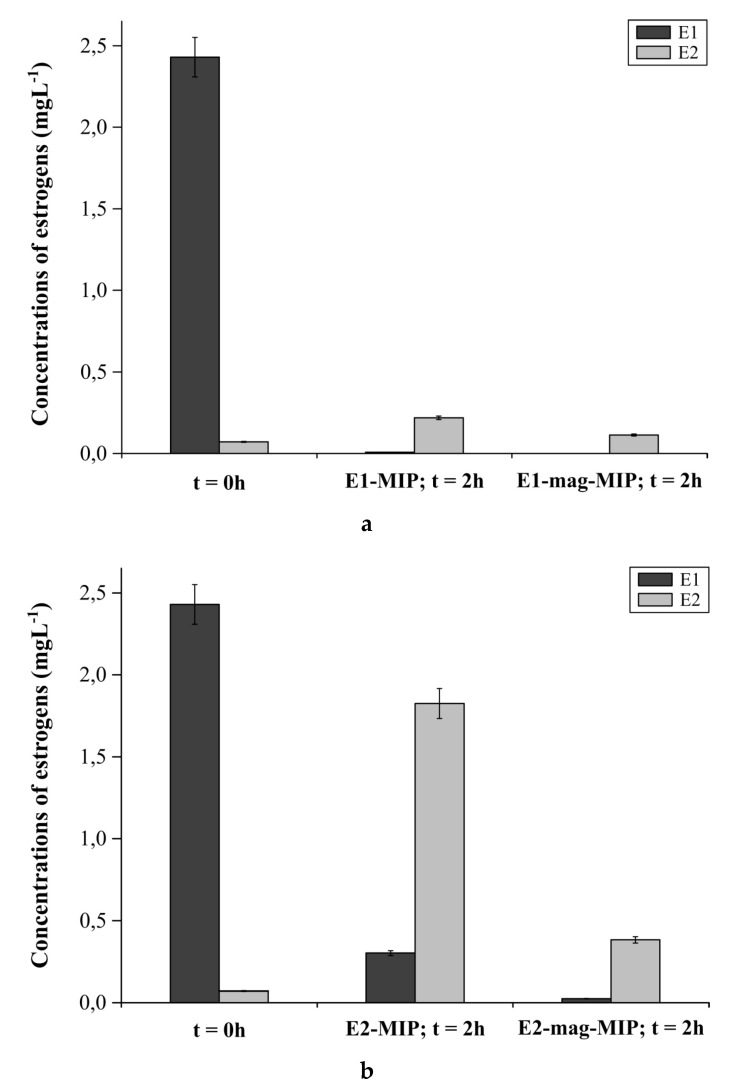
(**a**) Concentration of E1 and E2 in E1 aqueous solution before addition of the materials and 2 h after the addition of E1-MIP and E1-mag-MIP. (**b**) Concentration of E1 and E2 in E1 aqueous solution before addition of the materials and 2 h after the addition of E2-MIP and E2-mag-MIP.

The synthesis of two types of magnetic and non-magnetic MIP allowed the use of two different isolation procedures of the analyte: centrifugation and magnetic separation. However, the main goal was to compare the performance of the two materials. For this purpose, 10 mg of empty E1-MIP and 10 mg of empty E1-mag-MIP were added to two separate, 10 mL aqueous solutions of 2.7 mg L^−1^ E1. The concentration of E1 in water after the addition of the empty E1-MIP and E1-mag-MIP was examined after 2 h (Figure 6a).

Based on the amount of E1 in the solution after 2 h, in the samples to which E1-MIP and E1-mag-MIP were added, the sorption capacity of the materials was determined. E1-MIP bound 0.02 g of E1 per 1 g of the polymer. In comparison, while 1 g of a E1-mag-MIP bound 0.03 g of E1.

During the measurement at time t = 0 h for E1 solution, the low intensity signal assigned to E2 was observed. It is a result of the rapid conversion of E1 into the more stable E2 in the aqueous environment. Two hours after the addition of E1-mag-MIP, no signals from E1 in the solutions were detected, indicating the complete binding of the analyte to the selective cavities in the polymer structure. In the sample with E1-MIP, a low intensity signal, confirming the presence of small amounts of E1, was recorded. This confirms that the mag-MIPs adsorb analyte more efficiently. After 2 h, in both cases, the presence of E2 was found, indicating rapid conversion of E1 after the release from the polymer structure (as a result of the equilibrium state), occurring before the non-converted molecules were bound again. In the E1-mag-MIP solution, a signal of lower intensity was recorded. The detected signals from E2 indicate that it was not adsorbed within the E1-MIP and E1-mag-MIP structure, which confirms the selectivity of the obtained material.

To determine the selectivity of the polymers in relation to E1 and E2, the change in the concentration of E1 in water after addition of empty E2-MIP and E2-mag-MIP was examined (Figure 6b).

After 2 h of conducting the experiment, the E1 concentration decreased as a result of its inter-conversion into E2, which was successively bound by E2-MIP or E2-mag-MIP. E2-mag-MIP adsorbs newly formed E2 more quickly than E2-MIP. The state of chemical equilibrium shifts towards E2, and, as a result, E1 inter-converts faster in the sample with the addition of E2-mag-MIP. These studies confirmed that the polymers obtained have selective cavities for particular types of estrogens. In the first stage, E1 is transformed into E2, and, in the second stage, E2 is selectively adsorbed by E2-MIP and E2-mag-MIP.

**Figure 7:**
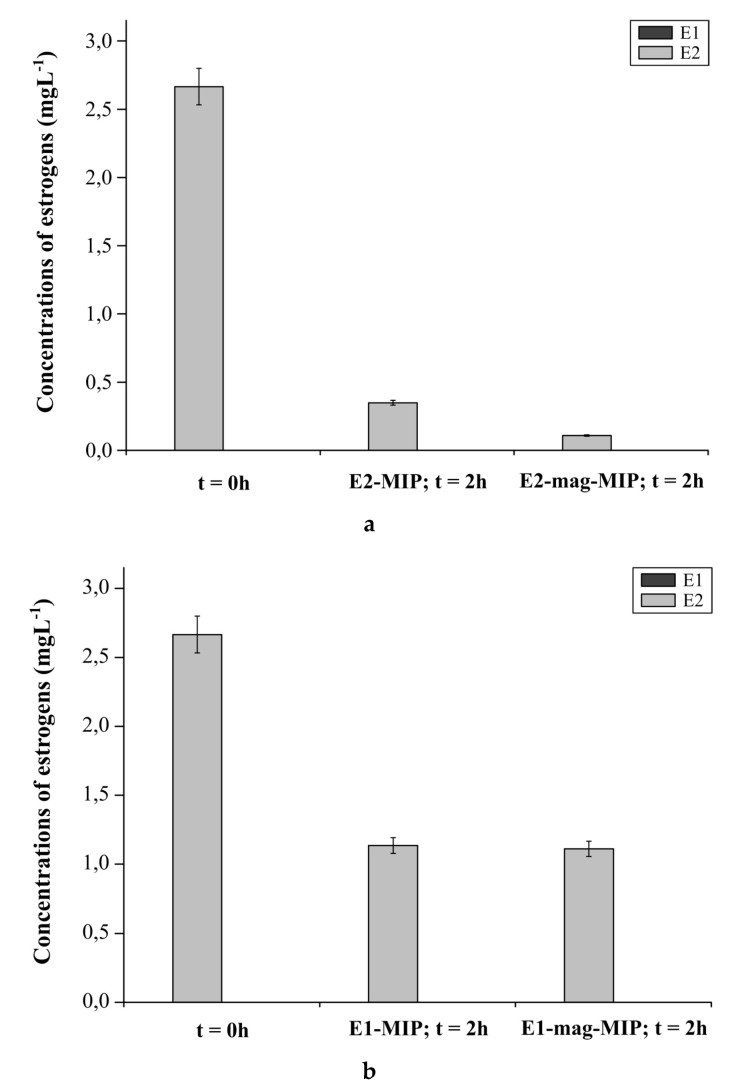
(**a**) Concentration of E1 and E2 in E2 aqueous solution before addition of the materials and 2 h after the addition of E2-MIP and E2-mag-MIP. (**b**) Concentration of E1 and E2 in E2 aqueous solution before addition of the materials and 2 h after the addition of E1-MIP and E1-mag-MIP.

The change in the concentration of E2 aqueous solution after addition of the empty E2-MIP and E2-mag-MIP after 2 h was also studied (Figure 7a).

Immediately after sample preparation, the signal for E2 was recorded. In this case, E1 was not found in the solution. After 2 h, the concentration of E2 significantly decreased in both samples. In the solution, E2-mag-MIP was observed to bind the analyte more efficiently than E2-MIP. Comparing the decrease in the concentration of E2 in the solution after the addition of E2-MIP and E2-mag-MIP, it was found that 1 g of E2-MIP bound 0.02 g of E2, while 1 g of E2-mag-MIP bound 0.03 g.

To determine the selectivity of the polymers towards E1 and E2, the change in the concentration of E2 in water after the addition of empty E1-MIP and E1-mag-MIP was examined (Figure 7b).

As E2 does not inter-convert into E1, only the signals from E2 were recorded. The decrease in the signal intensity after 2 h is associated with the rapid degradation in aqueous solutions, containing high concentration of the hormone. The loss of E2 after 2 h corresponds to the previously demonstrated hormone instability over time. E1-MIP and E1-mag-MIP do not absorb E2 in their E1 dedicated cavities. 

The empty E1-MIP/E1-mag-MIP (10 mg) added to the aqueous solution of E1 or E2-MIP/E2-mag-MIP added to the aqueous solution containing E2 in the concentration range of 0.027–27 μg L^−1^ resulted in binding of about 95% of the analytes within the first 5 min. The difference in the concentrations of the analytes is a measure of selectivity of the polymers. The selectivity of mag-MIPs, as measured by the amount of adsorbed estrogens, was E1/E2 of 100/10 for E1-mag-MIP and 100/5 for E2-mag-MIP.

The addition of the polymers with no selective cavities, NIP and mag-NIP, to the E1 and E2 water solutions caused no changes in the hormones’ concentrations, due to the lack of binding properties. The decrease in the hormones’ concentrations was associated with the process of their degradation in aqueous solutions. 

#### 3.2.6. The Effect of pH on the Estrogens Release from E1-Mag-MIP and E2-Mag-MIP

**Figure 8:**
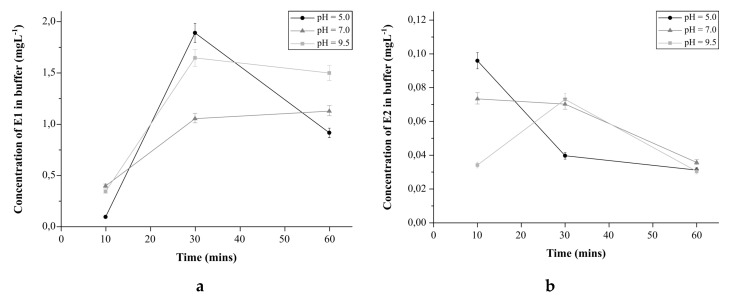
(**a**) The E1 release curve with E1-mag-MIP in solutions with different pH values: 5.0, 7.0, and 9.5. (**b**) The E2 release curve with E2-mag-MIP in solutions with different pH values: 5.0, 7.0, and 9.5.

To determine the stability of estrogens in mag-MIPs structures and the release kinetics of mag-MIPs analytes, their release was tested at various environmental pHs (Figure 8a,b).

After 10 min of running the experiment, E1 release from the polymer structure was low. The maximum concentration of E1 in all pH tested occurred after 30 min. The process of analyte recovery was most efficient at pH = 5. A characteristic feature of the release curves is that it reaches a plateau, as the maximum value is reached. However, not in this case. At pH = 5, E1 degradation was the fastest, which is unfavorable from an analytical point of view. At pH = 9.5, less E1 was released; however, degradation was much slower. At pH = 7, the smallest amount of analyte was released, which persisted over time, but did not give reliable quantitative results. For E1 analysis, pH plays a significant role. E1 is the most stable at pH = 7, but it is difficult to recover it from the polymer structure at this pH.

E2 release from E2-mag-MIP was much faster than E1 from E1-mag-MIP. Due to the minimal sample preparation time of 10 min after introducing E2-mag-MIP into buffer solutions, in two cases, maximum values of E2 concentration were recorded at the beginning of the measurement. At pH = 5, E2 was released at the highest concentration. At pH = 7, the amount of E2 released was lower. Regarding degradation rates, in an acidic environment, the concentration dropped rapidly, signifying the fastest degradation. At the neutral pH, E2 degradation occurred more slowly. At pH = 9.5, the maximum release of E2 from E2-mag-MIP occurred after 30 min, followed by degradation. The amount of E2 released was comparable to the amount of E2 released at pH = 7. In all samples, E2 degraded very quickly, which practically prevented its quantitative determination after release from the polymer matrix.

The estrogens bound in the structure of mag-MIPs are stable over time, and the release can be controlled by the means of pH or by temperature change after separation from the solution. In the aqueous solution, when E1 and E2 are released from MIP or mag-MIP, their chemical transformation and degradation takes place. The solution to this problem is direct analysis of estrogens from polymeric structures by the FAPA-MS method. 

#### 3.2.7. Analysis of Estrogens in an Aqueous Solution Using E1-/E2-Mag-MIP and FAPA-MS Technique 

**Figure 9:**
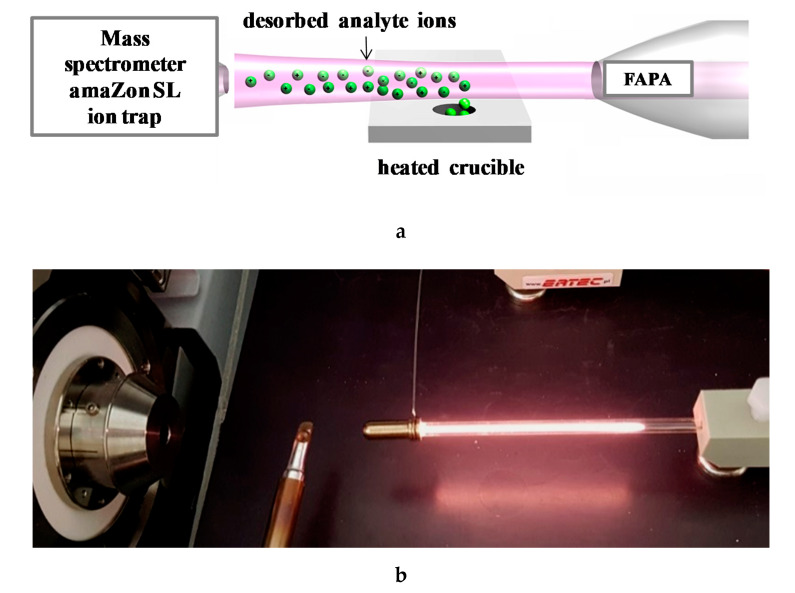
(**a**) Scheme the measuring system: FAPA ionization source, heated crucible, and amaZon SL mass spectrometer. (**b**) The measuring system: FAPA ionization source, heated crucible, and mass spectrometer inlet.

The analysis of estrogens was carried out using a plasma stream as an ionizing agent for the analytes. The scheme of the FAPA-MS measuring system is shown in Figure 9a,b.

**Figure 10:**
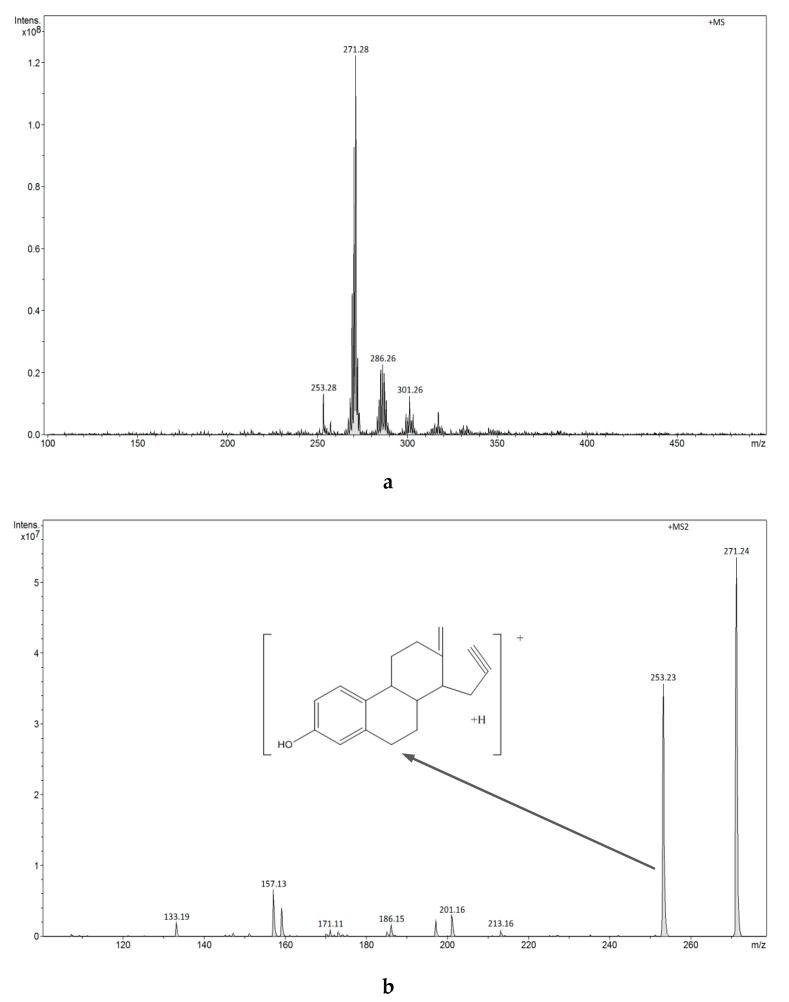
(**a**) FAPA-MS (positive ions) spectrum of E1. (**b**)FAPA-MS^2^ fragmentation spectrum of the *m/z* 271 ion observed for E1.

**Figure 11:**
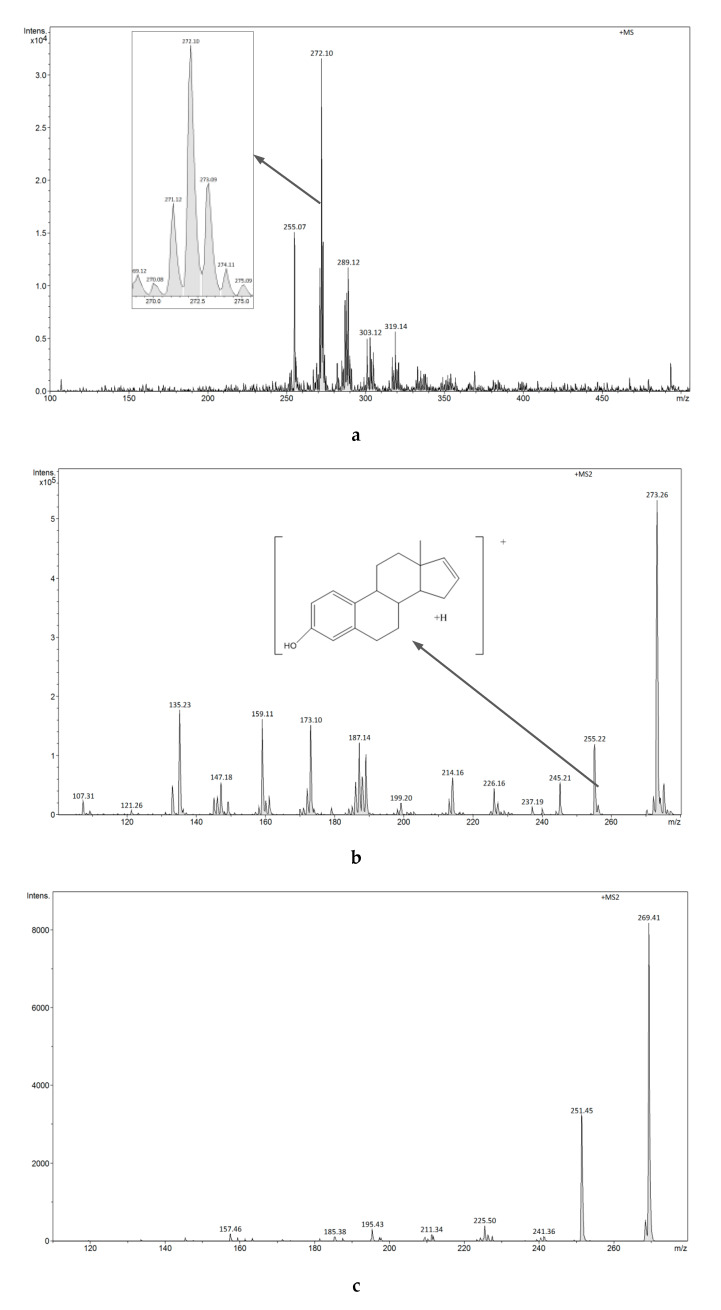
(**a**) FAPA-MS (positive ions) spectrum of E2. (**b**) FAPA-MS^2^ fragmentation spectrum of the *m/z* 273 ion observed for E2. (**c**) FAPA-MS^2^ fragmentation spectrum of the *m/z* 269 ion observed for E2.

The FAPA-MS spectra of estrogens using plasma ionization are shown in Figure 10a,b and Figure 11a–c. 

The FAPA-MS spectrum of E1 shows a signal at *m/z* 271 [M+H]^+^, whereas the FAPA-MS^2^ spectrum reveals the signals at *m/z* 133, *m/z* 157, *m/z* 197, and *m/z* 253 characteristic for E1 as a result of ion fragmentation *m/z* 271 [M+H]^+^. The FAPA-MS spectra of E1 using plasma ionization depend on the temperature of the heating table used for thermal desorption of the compound (260-300 °C). A signal with intensity changing from *m/z* 269 [M-2H]^+^ to 273 [M+H]^+^ is observed in the E2 spectrum, while the FAPA-MS^2^ spectrum reveals the signals at *m/z* 135, *m/z* 159, *m/z* 173, and *m/z* 255 characteristic of E2 as a result of ion fragmentation *m/z* 273 [M+H]^+^ and the signal at *m/z* 251 (Figure 11b) as a result of ion fragmentation *m/z* 269 [M+H]^+^ (Figure 11c).

**Figure 12:**
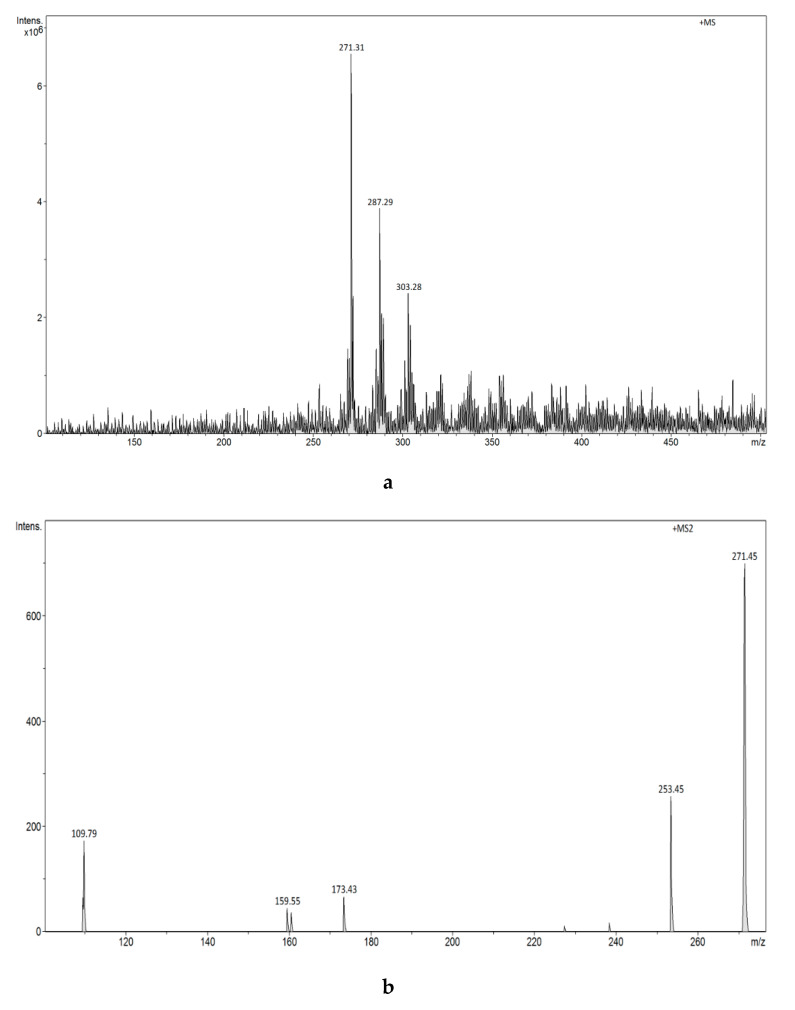
(**a**) FAPA-MS (positive ions) spectrum of E1 from E1-mag-MIP. (**b**) FAPA-MS^2^ fragmentation spectrum of the *m/z* 271 ion observed for E1 from E1-mag-MIP.

**Figure 13:**
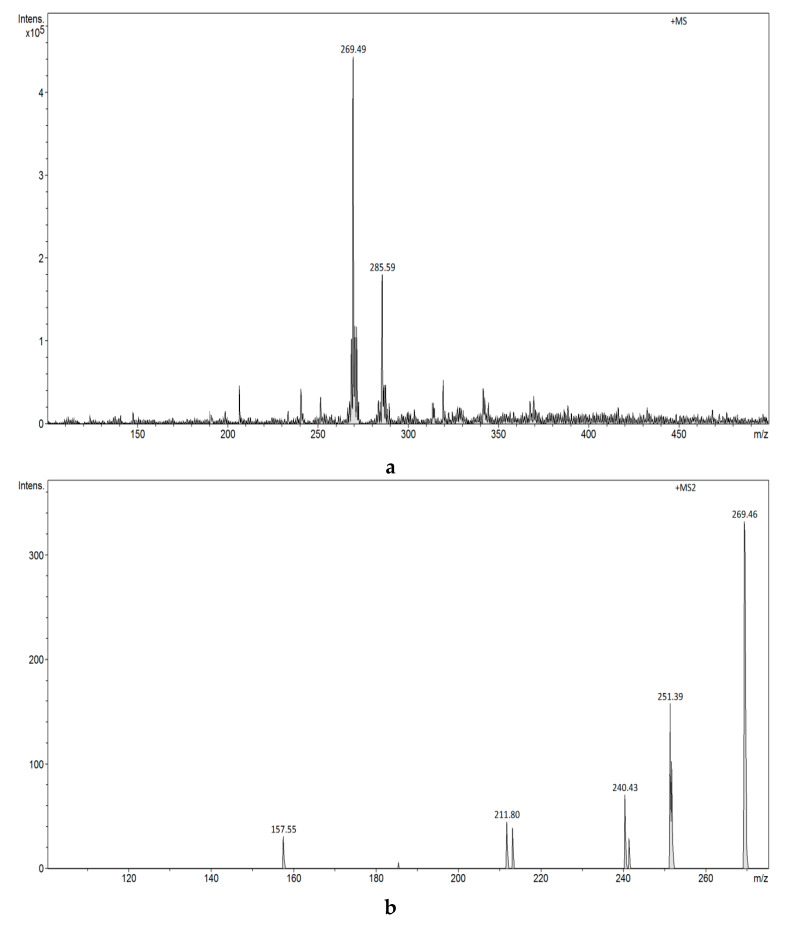
(**a**) FAPA-MS (positive ions) spectrum of E2 from E2-mag-MIP. (**b**) FAPA-MS^2^ fragmentation spectrum of the *m/z* 269 ion observed for E2 from E2-mag-MIP.

FAPA-MS analysis successfully detected the presence of E1 and E2 in samples. In addition, the source of FAPA ions utilizes mild ionization and provides less intense signals from fragmentation ions in comparison to ESI-MS. In the FAPA-MS^2^ spectra, the most intense fragmentation signal comes from the water molecule cleavage. In accordance with the procedure described in the experimental part Section 2.4.5, the obtained mag-MIPs were used to bind and concentrate estrogens occurring at low concentrations in water solutions and then to determine the estrogens content in polymers by means of thermal desorption and plasma ionization. The results are presented in Figure 12a,b and Figure 13a,b. 

The results of the conducted research show that the technique applied allows determination of estrogens in water at a concentration of 0.271 μg L^−1^ and that estrogens bound in MIPs/mag-MIPs do not undergo transformation in up to 72 h. This was confirmed by testing the material after 24, 48, and 72 h. The spectra were identical to the one obtained at T = 0. It was demonstrated that bound hormones are stable in polymeric structures, which enables their quantitative analysis and gives the possibility of stable storage of the analyte for a short time (three days). The method involving the use of mag-MIPs to concentrate, transport, and then determine estrogens by the FAPA-MS technique makes it possible to reduce the limit of detection of these compounds to 0.136 μg L^−1^ and allows measurements to be made during the time when the conversion of E1/E2 bound in mag-MIPs structures is inhibited. In addition, in the FAPA-MS technique, the analyte is directly and completely recovered from the polymer structure. This solves the problem related to the pH of the water sample, estrogen inter-conversion, and degradation.

## 4. Conclusions

A new, selective method for the determination of estrogens in aqueous solutions based on the use of MIP/mag-MIP dedicated to a given type of hormone is proposed. In addition, the FAPA-MS method was used as a modern tool enabling one-step direct testing of organic substances contained in the solid phase. The combination of this analytical technique with the molecular imprinting simplifies analytical pathway for the trace determination of the analytes in diluted solutions. In the case of hormone analysis, a combination of MIP/mag-MIP and FAPA-MS enabled selective analysis of E1 and E2, blocking their transformation and degradation, by closing the analytes in selective polymer structures, allowing a direct quantitative analysis from the solid phase. It has been proven that both types of polymers can be used successfully, but the polymers with magnetic properties show better performance in the analytical process. The scope of this method was established, and the results obtained were compared with those obtained with the classical ESI-MS method. Due to the combined approach of molecular imprinting techniques and the innovative analytical FAPA-MS method, a new, fast, and effective method for the selective determination of estrogens is proposed.

## Supplementary Materials

The following are available online at https://www.mdpi.com/2218-273X/10/5/672/s1, Table S1: Physicochemical properties of natural steroidal estrogens, Figure S1: The steps for the preparation of estrogens (R = O or -OH) molecularly imprinted polymers, Figure S2: SEM images of the obtained polymers, Figure S3: ESI-MS (positive ions) spectrum of E1, Figure S4: Fragmentation spectrum of the *m/z* 271 ion observed for E1, Figure S5: ESI-MS (positive ions) spectrum of E2, Figure S6: Fragmentation spectrum of the *m/z* 273 ion observed for E2, Figure S7: Dependence of signal intensity vs. estrogens concentration in the sample.
