# Supplementary material for: Molecularly Imprinted Polymers and Magnetic Molecularly Imprinted Polymers for Selective Determination of Estrogens in Water by ESI-MS/FAPA-MS
Source: Biomolecules. 2020 Apr 27;10(5):672. doi: 10.3390/biom10050672 (PMC7277882; doi:10.3390/biom10050672)
Supplement: Supplementary file 1 [file biomolecules-10-00672-s001.pdf]

Supplementary Materials

Molecularly imprinted polymers and magnetic molecularly imprinted polymers for selective determination of estrogens in water by ESI-MS/FAPA-MS

Maria Guć [1]* and Grzegorz Schroeder [1]

[1] Faculty of Chemistry, Adam Mickiewicz University in Poznań, Uniwersytetu Poznańskiego 8, 61-614 Poznań, Poland; maria.guc@amu.edu.pl, schroede@amu.edu.pl

* Correspondence: maria.guc@amu.edu.pl

Table 1. Physicochemical properties of natural steroidal estrogens.

Name and symbol	Structural formula	Molecular formula	Molecular weight [g mol^{-1}]	Water solubility [mg L^{-1}] in 25°C	Melting point [°C]
Estron (E1)	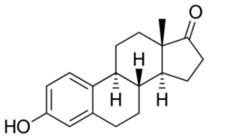	$C_{18}H_{22}O_2$	270.37	0.8-30.00	260
Estradiol (E2)	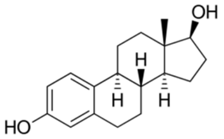	$C_{18}H_{24}O_2$	272.38	1.51-12.96	178
Estriol (E3)	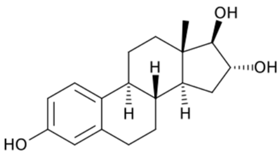	$C_{18}H_{24}O_3$	288.38	1.38-27.34	282

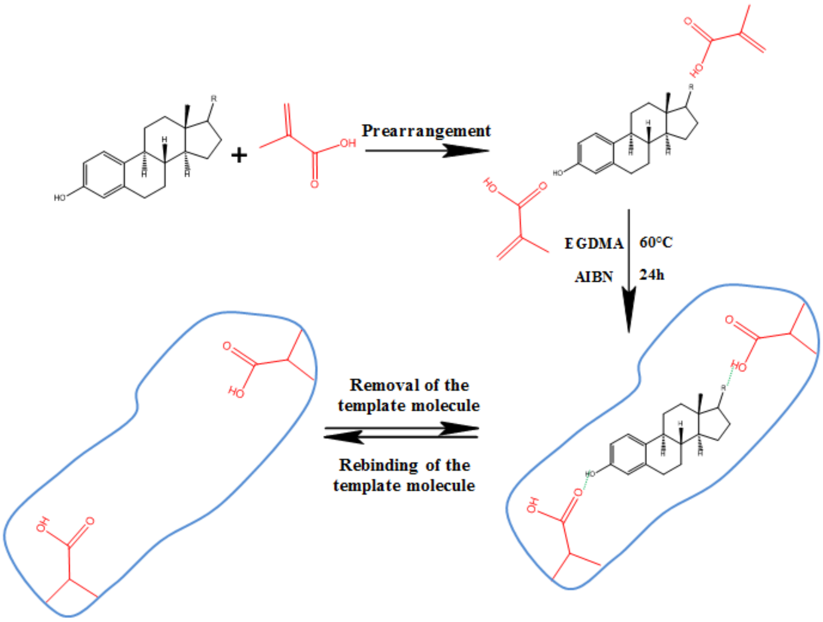

Figure S1. The steps for the preparation of estrogens (R = O or OH) molecularly imprinted polymers.

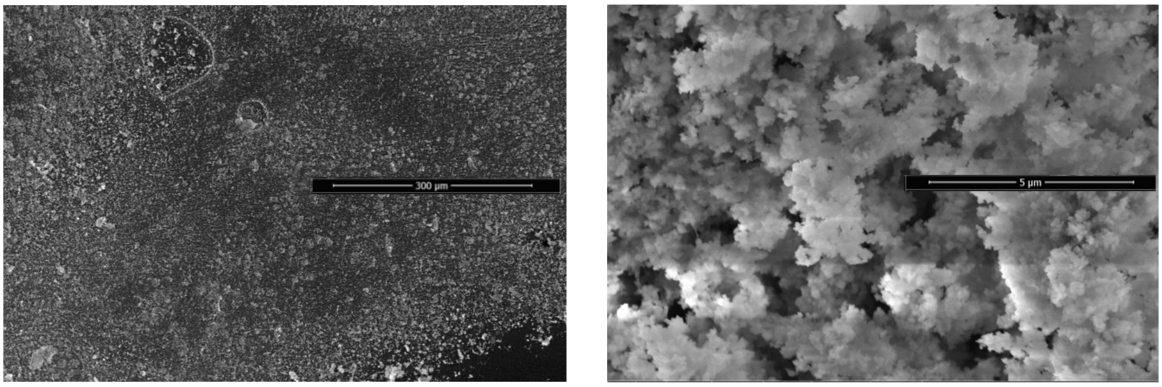

(a) E1-MIP

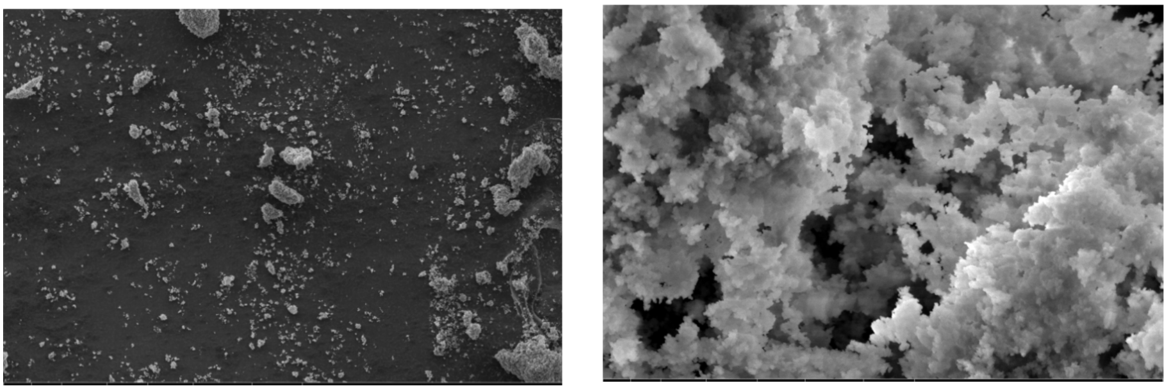

(b) E1-MIP after washing of the template

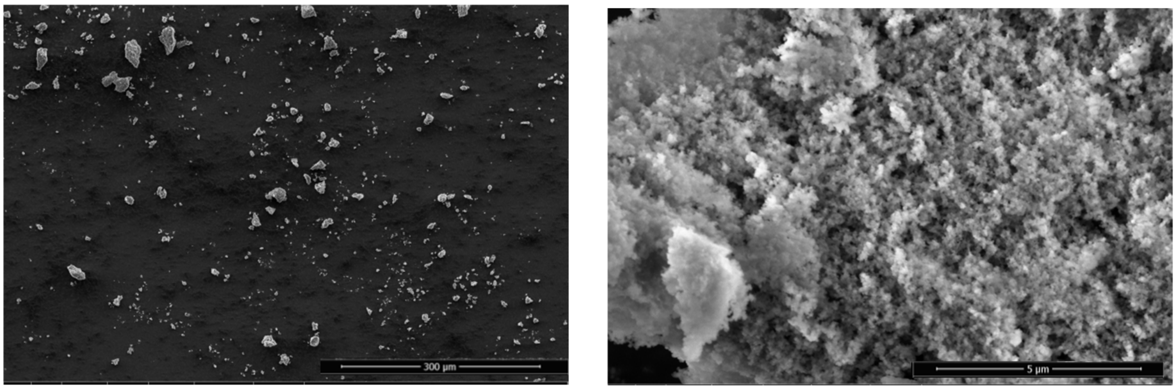

(c) E1-NIP

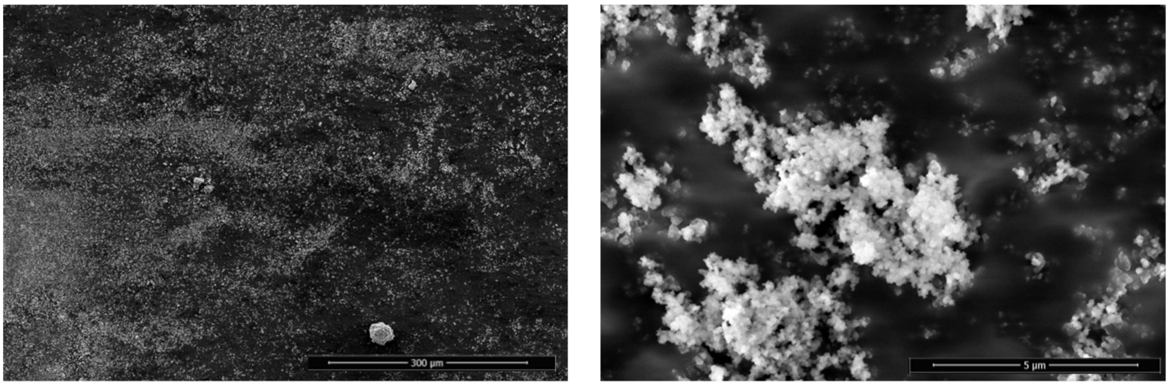

(d) E2-MIP

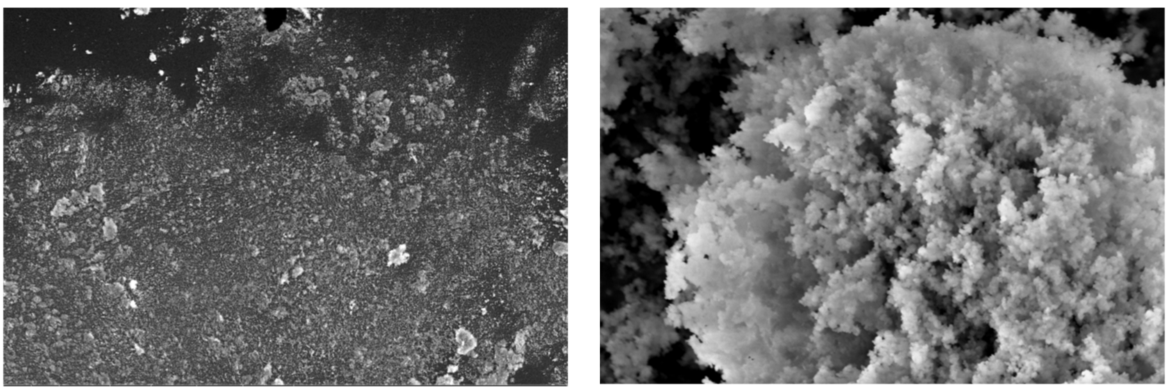

(e) E2-MIP after washing of the template

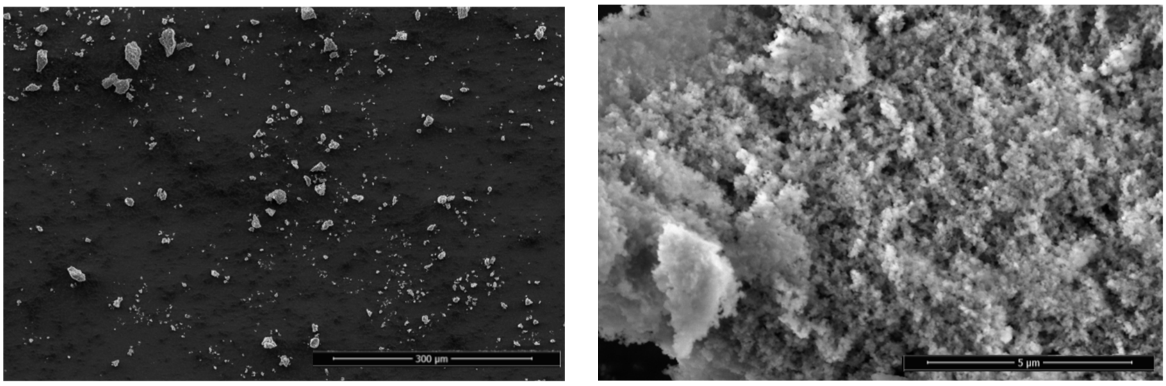

(f) E2-NIP

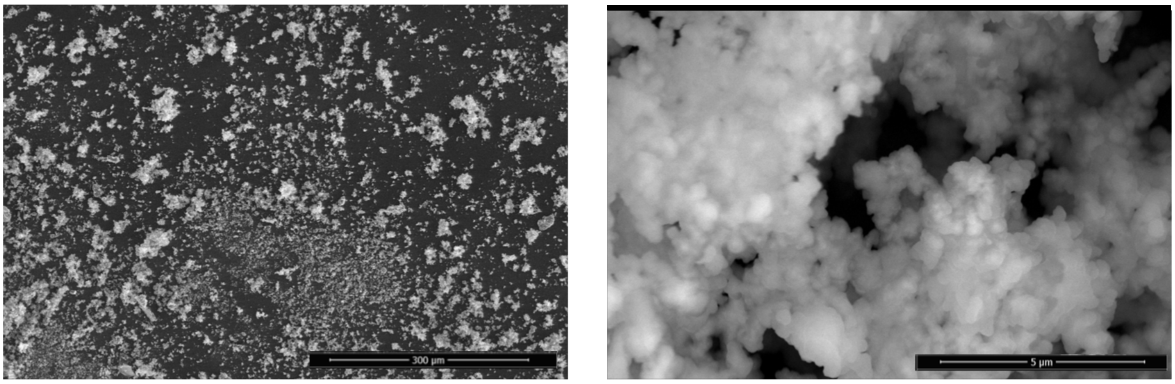

g) E1-mag-MIP

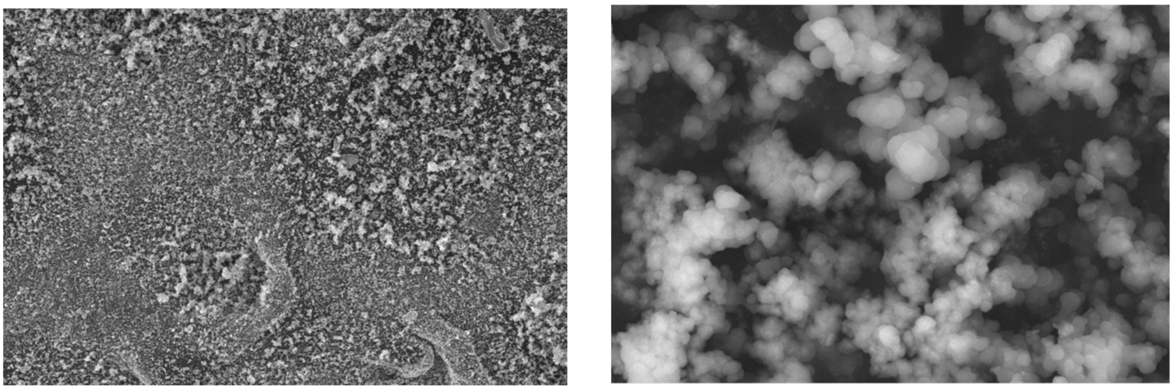

(h) E1-mag-MIP after washing of the template

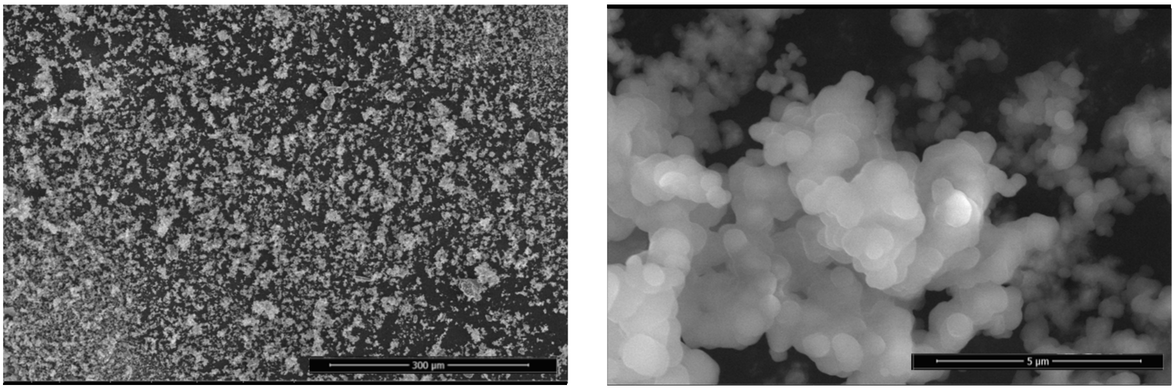

(i) E1-mag-NIP

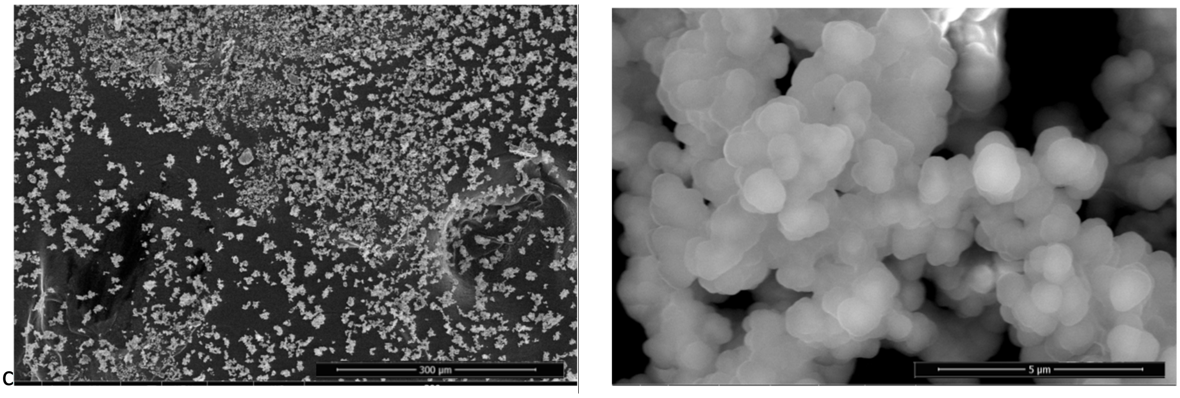

(j) E2-mag-MIP

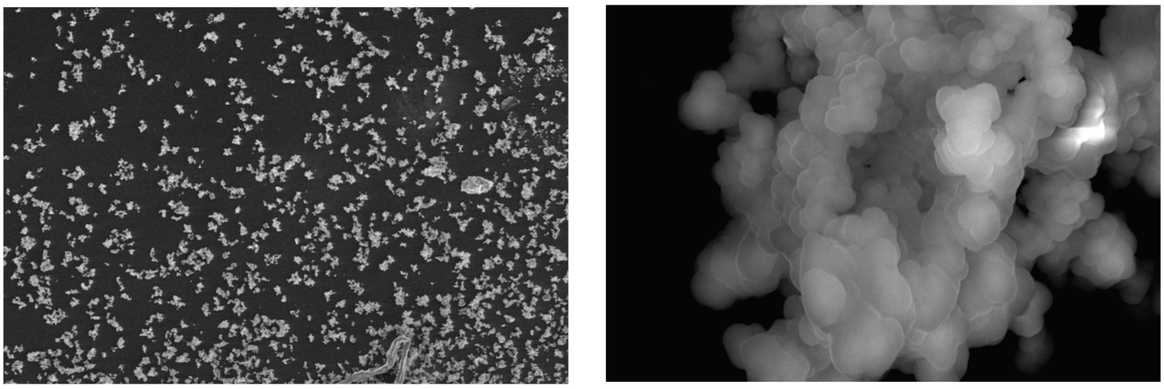

(k) E2-mag-MIP after washing of the template

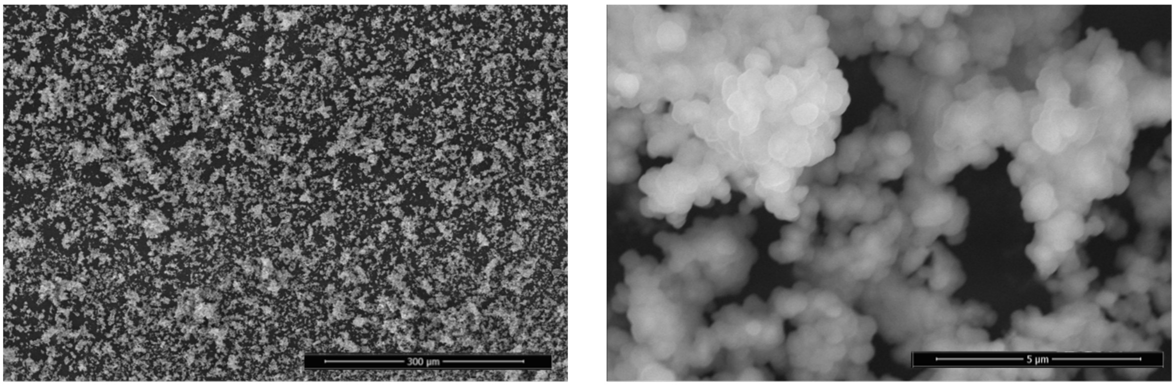

(l) E2-mag-NIP

Figure S2. SEM images of the obtained polymers.

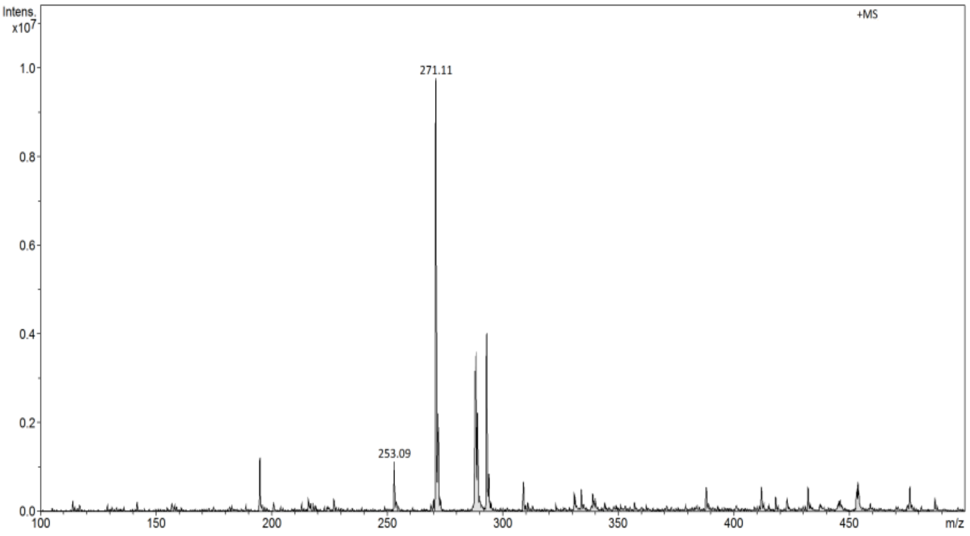

Figure S3. ESI-MS (positive ions) spectrum of E1.

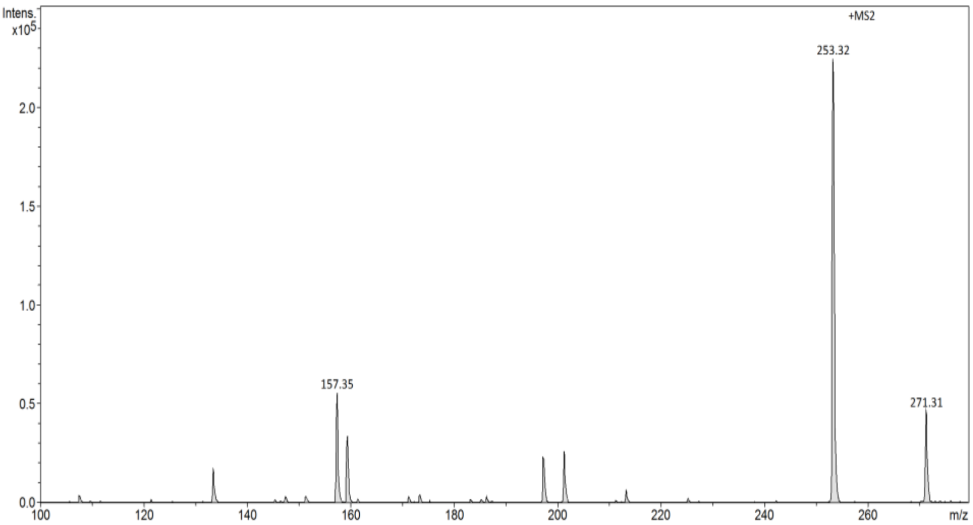

Figure S4. Fragmentation spectrum of the m/z 271 ion observed in E1.

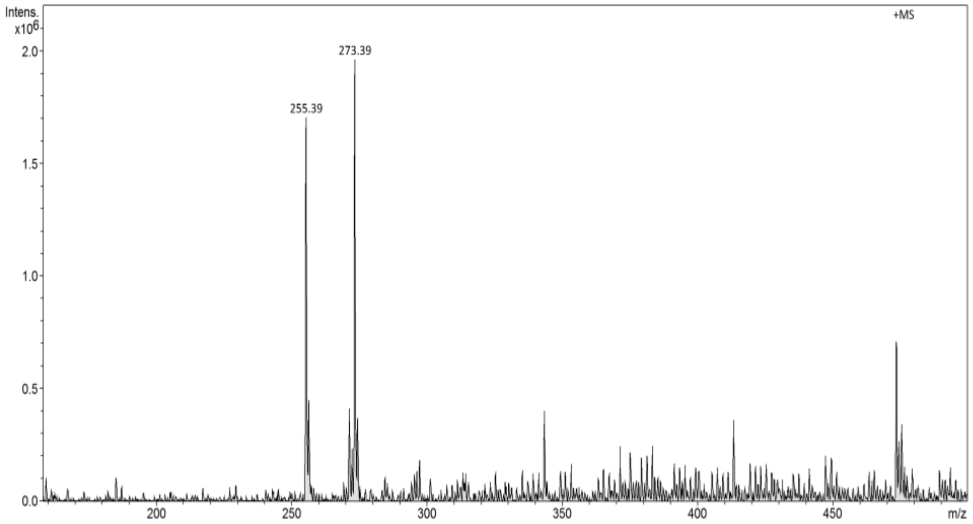

Figure 5. ESI-MS (positive ions) spectrum of E2.

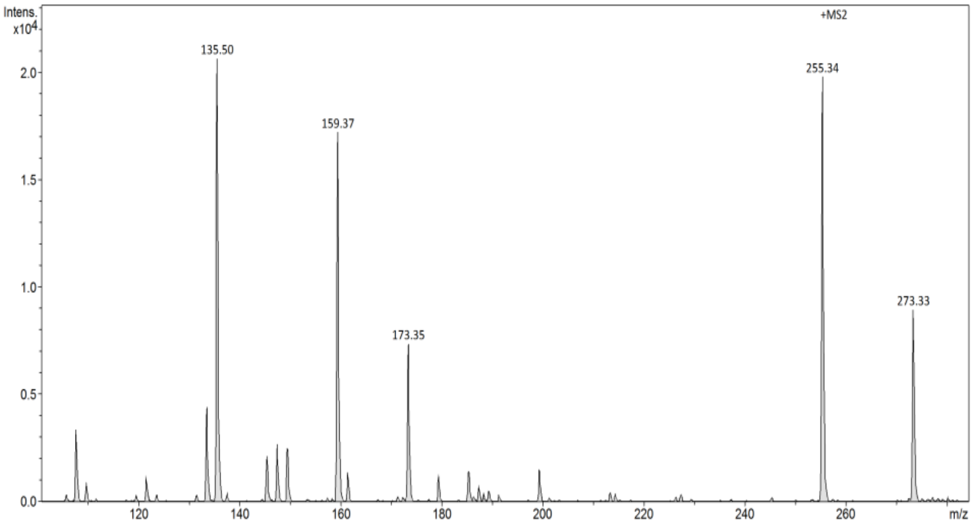

Figure S6. Fragmentation spectrum of the m/z 273 ion observed in E2.

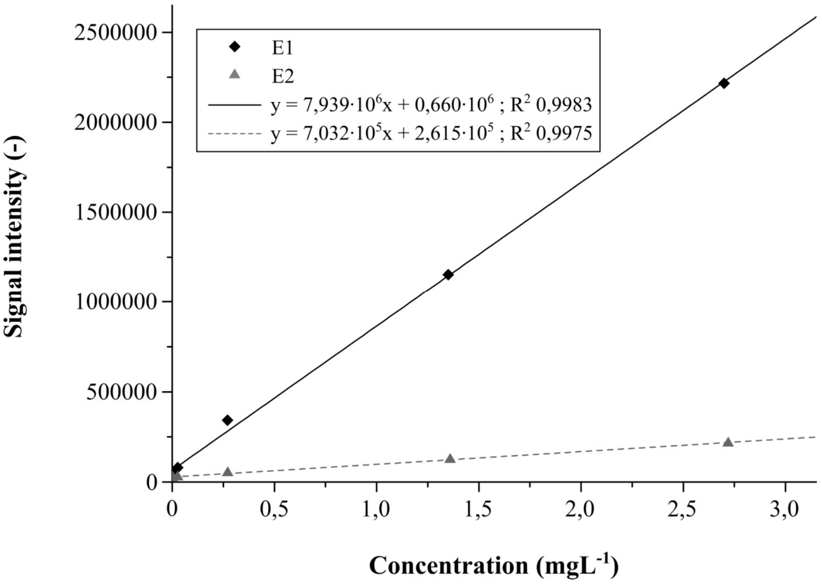

Figure S7. Dependence of signal intensity *vs.* estrogens concentration in the sample.

.
